# Deep neural networks in real-time coherent diffraction imaging

**DOI:** 10.1107/S2052252520016590

**Published:** 2021-01-01

**Authors:** Ross Harder

**Affiliations:** a Advanced Photon Source, Argonne National Laboratory, Argonne, IL 60439, USA

**Keywords:** machine learning, Bragg coherent X-ray diffraction, phase retrieval, single-particle imaging, deep neural networks

## Abstract

The potential for convolutional neural networks to provide real-time imaging capabilities for coherent diffraction imaging experiments at XFELs is discussed.

X-ray imaging methods have progressed rapidly at modern synchrotron light sources owing to the highly collimated, bright, tunable X-ray beams available at them. Included in these advances are high-speed micro-tomography and nanoscale three-dimensional imaging with computed tomography (Maire & Withers, 2014 ▸).

In parallel, coherent imaging methods have become a powerful tool for nanoscale imaging of material and biological structure. When combined with tomography and scanning techniques they can achieve quantitative determination of density (Diaz *et al.*, 2015 ▸). When used in conjunction with Bragg diffraction one gains sensitivity to crystalline structure, including strain and deformations due to external stimuli (Pfeifer *et al.*, 2006 ▸).

In the Bragg diffraction case, the technique can struggle to be effective in some cases. As pointed out by Wu and coworkers in this issue of **IUCrJ** (Wu *et al.*, 2021 ▸), highly strained crystals and samples composed of many interfering domains can produce coherent diffraction patterns that are difficult to invert. In addition to this occasional struggle when implementing the method, fourth generation X-ray facilities such as X-ray free electron lasers (XFELs) and diffraction-limited storage rings can generate a flood of coherent imaging data (Sobolev *et al.*, 2020 ▸).

Coherent imaging methods use measurements in reciprocal space, where coherently interfering beams produce an interference pattern on the detector. In the case of Bragg coherent imaging the data are acquired in the vicinity of a Bragg peak of the lattice, where typically a volume of reciprocal space is recorded to enable 3D imaging in real space, though 2D imaging from just a single slice through reciprocal space is also possible.

Central to the success of coherent imaging has been the development of iterative phase retrieval algorithms (Fienup, 1982 ▸). These computational tools invert the reciprocal space interference patterns, or diffraction patterns, to an image of the sample in real space. Generally based on both forward and inverse discrete Fourier transforms and iterated in both directions, these algorithms see varied success at converging to a reliable solution to the phase problem, sometimes depending on many algorithmic parameters and expert methods (Clark *et al.*, 2015 ▸). They also rely heavily on a fine sampling of the interference pattern in reciprocal space. As a result, the computational arrays can be large, both due to high sampling requirements in the data and the resulting over-determination of real space extents (finite support) of the object where it will be surrounded by zeros in the computational array. Since the computational cost of discrete Fourier transforms scales as the logarithm of the size of the array, one quickly reaches relatively long computational times and extensive memory requirements to retrieve an image. These computational requirements are daunting, particularly in the face of the current and future fourth generation X-ray sources like free electron lasers and diffraction-limited storage rings. At these facilities one can produce 2D coherent diffraction data at kilohertz rates or sample large 3D volumes of reciprocal space in tens of seconds.

The current computational requirements of coherent imaging, and other imaging methods, have led scientists to explore the use of modern data analytical techniques to solve problems in image analysis and reconstruction. Machine Learning (ML) methods and in particular the Convolutional Neural Network (CNN), are being used to tackle diverse challenges in X-ray data analysis, including limited angle and streaming tomography (Huang *et al.*, 2020 ▸; Liu, Z. *et al.*, 2019 ▸), and determination of space groups directly from pair distribution function data (Liu, C.-H. *et al.*, 2019 ▸). For coherent diffraction imaging, the idea (see Fig. 1 ▸) is quite simple, can a machine be trained to simply look at a coherent diffraction pattern and create, in its synthetic mind, an image of the sample? While using only modest ML tools, Wu *et al.* have shown that the answer to this question may be ‘Yes’. The key to the success of using CNNs to invert coherent diffraction patterns lies in the extent of the training data used to form the neural network. Effectively, the use of a CNN moves the bulk of the computational cost far ahead of the experiment. Using an extensive set of images and corresponding simulated diffraction patterns, the neural network is trained to build the connection between reciprocal and real space into the neural encoding.

Working in 2D with the goal to provide real-time inversion in XFEL ultrafast time resolved imaging experiments, Wu *et al.* have trained a so-called encoder–decoder CNN. Here a set of diffraction patterns have been used to develop a single encoding which is then decoded to produce both the shape and inherent deformation (encoded as phase of input complex density input into the training) of the crystal lattice (Fig. 2 ▸). Using additional simulated data to test the trained CNN they report 0.5 ms inversion time! For experimental data they found that a small number of iterations of traditional phase retrieval algorithms were required to converge to a reliable image. This was done, however, with expert-free requirements and found to be highly robust. The result of this work, and other ongoing research in the field, is that the throughput of CDI phase retrieval could keep pace with flood of data at modern instruments (Cherukara *et al.*, 2018 ▸, 2020 ▸; Scheinker & Pokharel, 2020 ▸). These advances also enable the expansion coherent imaging applications into scientific realms where current inversion methods either fail or require extensive expertise.

With single-particle coherent scattering experiments at XFELs starting to reach significant hit rates for MHz X-ray pulse frequencies, the potential for CNNs to provide real-time imaging capabilities is just being touched. As researchers continue to expand and improve on the ML implementations, we can expect the impact of work like that of Wu and coworkers to bear significant fruit for high-resolution X-ray imaging.

## Figures and Tables

**Figure 1:**
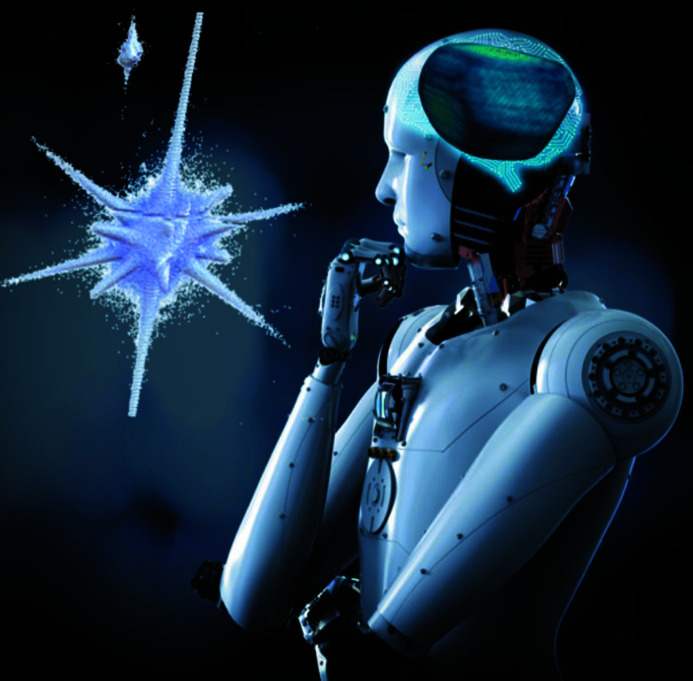
A machine inspects a coherent diffraction pattern and directly forms an image of the sample in its mind. Adapted from Shutterstock image 732725557 by Phonlamai. https://www.shutterstock.com/image-illustration/3d-rendering-artificial-intelligence-brain-ai-732725557

**Figure 2:**
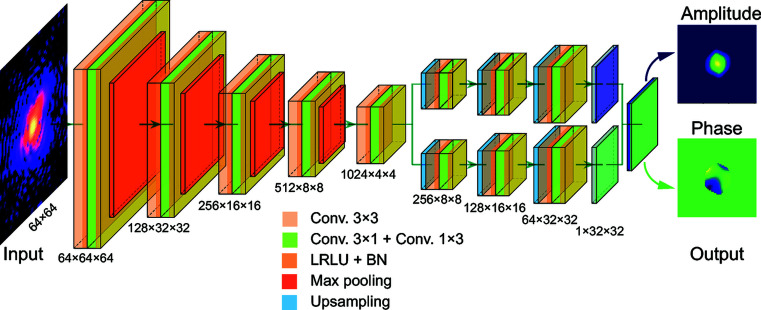
A schematic visualization of the deep neural network for single-particle-imaging inversion used by Wu *et al.* (2021 ▸). The neural network is implemented using an architecture composed entirely of convolutional, maximum pooling and upsampling layers. In the network, Conv. refers to convolution, LRLU refers to the leaky rectified linear unit and BN refers to batch normalization.
